# Socioeconomic inequality in organized and opportunistic screening for colorectal cancer: results from the Korean National Cancer Screening Survey, 2009-2021

**DOI:** 10.4178/epih.e2023086

**Published:** 2023-09-17

**Authors:** Xuan Quy Luu, Kyeongmin Lee, Jae Kwan Jun, Mina Suh, Kui Son Choi

**Affiliations:** 1 Department of Cancer Control and Population Health, Graduate School of Cancer Science and Policy, National Cancer Center, Goyang, Korea; 2 National Cancer Control Institute, National Cancer Center, Goyang, Korea

**Keywords:** Colorectal cancer, Mass screening, Health inequities, Healthcare disparity

## Abstract

**OBJECTIVES:**

This study aimed to investigate socioeconomic status (SES)-based inequality in colorectal cancer (CRC) screening in Korea. We assessed whether the rates of opportunistic and organized CRC screening differed according to income and education levels.

**METHODS:**

We analyzed data from the Korean National Cancer Screening Survey of 27,654 cancer-free individuals, aged 50-74 years, from 2009 to 2021. The weighted cancer screening rates with trends were estimated with the average annual percentage change using joinpoint regression. Inequality was calculated in both relative and absolute terms, based on a Poisson regression model.

**RESULTS:**

The organized screening rate increased significantly from 22.1% in 2009 to 53.1% in 2020 and 50.6% in 2021, with an average annual change of 8.6% (95% confidence interval [CI], 4.9 to 12.5). In contrast, no significant trend was observed for opportunistic screening. The SES inequality in opportunistic screening uptake was indicated by a slope index of inequality (SII) of 9.74% (95% CI, 6.36 to 13.12), relative index of inequality (RII) of 2.18 (95% CI, 1.75 to 2.70) in terms of education level; and an SII of 7.03% (95% CI, 4.09 to 9.98), RII of 1.81 (95% CI, 1.41 to 2.31) in terms of measured income. Although there was an increasing trend in income inequality, no significant SES inequalities were observed in the overall estimates for organized screening.

**CONCLUSIONS:**

Organized CRC screening is effective in improving the participation rate, regardless of SES. However, significant inequalities were found in opportunistic screening, suggesting room for improvement in the overall equity of CRC screening.

## GRAPHICAL ABSTRACT

[Fig f4-epih-45-e2023086]

## INTRODUCTION

Colorectal cancer (CRC) is the third most common malignancy, with an estimated 2 million new cases and nearly 1 million deaths reported in 2020 [1]. Projections suggest that the number of new CRC cases will exceed 3 million by 2040 [2]. Although the highest burden of CRC has been observed in developed regions such as Europe, North America, and Australia, Asian countries account for more than half of CRC cases and deaths [1]. The burden of CRC tends to be more pronounced among individuals of lower socioeconomic status (SES), due to differences in risk factors and access to cancer prevention services [3]. In Korea, the incidence of CRC has increased over recent decades [4], and in 2018, the country reported the highest incidence of CRC among male worldwide [5]. This trend in the CRC burden in Korea is largely attributed to a Westernized lifestyle characterized by an unhealthy diet and low levels of physical activity [6]. Furthermore, the increasing number of reported cases is linked to the expansion of screening services in Korea.

Many countries have introduced both organized and opportunistic CRC screening to reduce the cancer burden. However, organized screening and other public health interventions may either reduce or exacerbate SES inequalities [7,8]. With organized screening, individuals of lower SES can access CRC screening with partial or full financial assistance. Nonetheless, numerous studies have reported lower participation rates among less-deprived groups [3,9-12], resulting in a widened disparity in the CRC burden. It is crucial to carefully monitor information regarding CRC screening among various SES subgroups to ensure that appropriate interventions are implemented to reduce SES inequality. In Korea, the National Cancer Screening Program (NCSP) introduced CRC screening in 2004, utilizing a fecal immunochemical test (FIT) for males and females aged 50 or older [13]. Colonoscopy is used to further investigate FIT-positive individuals in the NCSP group. Otherwise, individuals have the ability to undergo opportunistic screening, for which they bear the financial responsibility. In the case of opportunistic screening, colonoscopy is widely accessible across the country at a cost that is more affordable than in other developed countries [14,15]. Recently, there have been reports of improvements in CRC screening rates, and the effectiveness of CRC screening has gained widespread acceptance in Korea [11,13,16,17]. However, there seems to be insufficient consideration as to whether CRC screening is being evenly distributed among all individuals.

Therefore, we investigated SES-based inequalities in CRC screening in Korea. Specifically, we assessed whether the screening rate of CRC, including both opportunistic and organized screening, differed according to individual income and education levels.

## MATERIALS AND METHODS

### Study material

The Korean National Cancer Screening Survey (KNCSS) is an annual, nationally representative survey designed to examine screening rates and related issues in Korea [13]. The primary target demographic for the KNCSS includes cancer-free males and females, aged 40 years to 74 years and 20 years to 74 years respectively, in accordance with the protocol for the five common cancer sites included in the NCSP. In the initial years of the KNCSS, the survey sample size was roughly 2,000, but this was increased to 4,100 in 2010 and further expanded to 4,500 in 2014. The KNCSS employs a stratified, multistage sampling method, which is based on the geographical area, age, and sex distribution of the Korean population. Participants were recruited through door-to-door contact, with a minimum of 3 attempts made to reach each household. One individual was selected from each household to participate. Before their involvement in the survey, all participants were given thorough explanations about the study’s purpose, procedures, and expectations, and voluntary consent was obtained. Data were collected through face-to-face interviews conducted by a professional research agency. Detailed information about the sampling methods can be found in previous studies [13,18]. Survey weights were employed to adjust the relative influence of individual units contributing to the overall population estimate.

As the main focus of the current study was CRC screening, we included only individuals 50-74 years old, according to the NCSP protocol [13]. Although the first KNCSS was conducted in 2004, the survey instrument was revised to reflect changes in the NCSP protocol and screening recommendations. Therefore, this study analyzed data from 2009 to 2021 to ensure maximum consistency in the measurement of the variables of interest.

### Measurements

The screening status and type for CRC were determined using a series of questions. These included: “Have you ever undergone CRC screening?”, “Which screening method have you experienced?”, “When did your most recent screening take place?”, and “How did you finance your CRC screening?”. Individuals who had undergone a FIT within the past year or a colonoscopy within the past ten years were classified as screened individuals. These screened participants were further categorized based on their payment source. If the national health insurance or government covered the cost, the screening was classified as organized. If not, it was classified as opportunistic. Individuals who underwent both types of screening were included in each type of screening.

Our study considered several key socio-demographic factors, including age, sex, residential area, educational level, and household income. The educational level was divided into four categories: those who completed elementary school or lower, middle school, high school, and college/university or higher. In the KNCSS, household income was divided into 13 groups, with a range from approximately 1,000 US dollar (USD) to 10,000 USD or more. However, the relatively small populations in some of these categories could potentially skew the overall estimation of inequality when the data is analyzed by screening type. To mitigate this, we reorganized and reclassified household income into three groups: low-income, middle-income, and high-income. This reclassification was based on the tertile distribution of the sample, ensuring a balanced representation of households across income groups. As the SES of the country has evolved over time, we applied different cut-off points based on the distribution of income variables. The income level was divided into the following sub-groups: <1,500, 1,500-2,999, ≥ 3,000 in 2009; < 2,000, 2,000-3,499, ≥ 3,500 in 2010 to 2012; and < 3,000, 3,000-4,499, ≥ 4,500 in 2013 to 2021.

### Statistical analysis

The organized and opportunistic screening rates for CRC were as the weighted screening rate for each year of the survey, spanning from 2009 to 2021. We used joinpoint regression to assess trends in the screening rate, using the weighted screening rate. This rate was then fitted into the model, resulting in the best-fit line. Depending on the data pattern over time, this line could be either single or multi-segmented [19,20]. Subsequently, we reported the annual percentage change (APC) for each segment and the average annual percentage change (AAPC) over the 13-year period from 2009 to 2021, both with a 95% confidence interval (CI).

For a comprehensive understanding of inequality in CRC screening, our study employed both the slope index of inequality (SII) and the relative index of inequality (RII) as representative measures of absolute and relative inequality, respectively [8,21]. These indices are population-weighted, regression-based measures, defined by the slope of the regression line, which illustrates the relationship between a group’s health and its relative socioeconomic rank [21]. They represent the shifts in health behavior from the most deprived individuals to the least, adjusting for the distribution of SES across the entire population. The SII is an absolute measure of the difference in health outcomes between the least and the most socioeconomically deprived individuals in the population. Therefore, a positive SII indicates a higher CRC screening rate in the least deprived group compared to the most deprived group, while a negative SII suggests the opposite. In contrast, the RII measures the ratio of the value for the most deprived category (reference) to the value for the least deprived, thus reflecting relative disparity. An RII value greater than 1 indicates a fold change in the screening rate of the highest SES group compared to the lowest, and vice versa. Inequality indices were estimated by ranking the education and income groups and assigning a “ridit” score to each category. This score was calculated by identifying the midpoint within the cumulative distribution range for each group. The “RIIGEN module” in Stata was used for this purpose [22]. The SII was derived from the slope of the weighted least squares regression, and the RII was estimated by dividing the SII by the mean of the population health condition/issue [23]. Both indices were calculated using the Poisson regression model of CRC screening in relation to educational level and income, with additional adjustments for age, sex, and residential area. Finally, the overall (pooled) estimates of the SII and RII over the 13-year study period were also calculated.

Descriptive analysis statistics and the estimation of inequality indices were conducted using Stata version 16 (StataCorp., College Station, TX, USA). The joinpoint regression analysis was carried out using the Joinpoint Regression Program version 4.9.1.0 (National Cancer Institute, Bethesda, MD, USA). A p-value of less than 0.05 was considered statistically significant.

### Ethics statement

This study was approved by the Institutional Review Board of the National Cancer Center, Korea (IRB No. NCC-2019-0233). The written informed consent was waived due to public purposes.

## RESULTS

### Characteristics of the study population

**Figure 1.**
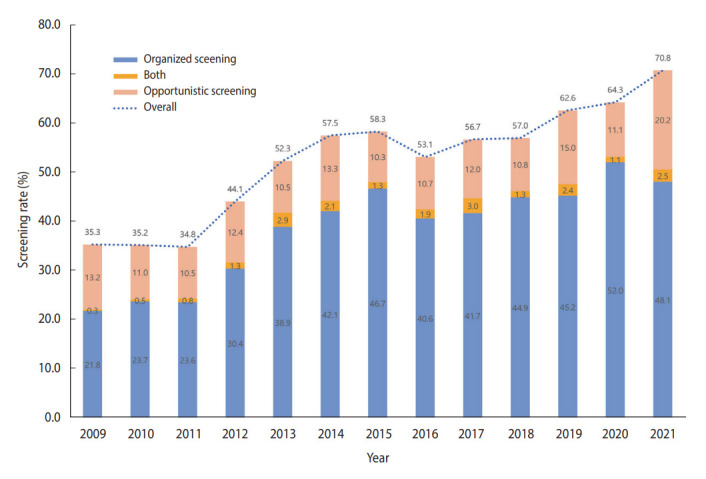
Screening rate for colorectal cancer by screening type from 2009 to 2021.

In total, 27,654 cancer-free individuals aged 50-74 years who participated in the KNCSS between 2009 and 2021 were included in the final analysis (Table 1). Except for 2009, which had 937 participants, the survey population ranged from approximately 2,000 individuals to 2,500 individuals. Overall, males accounted for a slightly smaller proportion than females, and approximately 50% of the study population were 50-59 years old in all survey years (Table 1). The overall-screening and screening rate by type of screening over 13 years are shown in Figure 1. The screening rate for CRC, following the recommendation, saw a significant increase from about 35% in 2009 to nearly double by 2021. Organized screening was responsible for the majority of CRC screenings (Figure 1).

Generally, the organized screening rate increased significantly, from only 22.1% in 2009 to 53.1% in 2021 and 50.6% in 2021, with an AAPC of 8.6% (95% CI, 4.9 to 12.5) (Table 2). The sharpest increase in the organized screening rate was observed in the period from 2009 to 2013 (APC= 21.64%, p<0.001), followed by a slight increase in later years (Supplementary Material 1). While a similar increasing trend was observed in all socio-demographic subgroups, a higher AAPC was observed among people who lived in non-metropolitan areas, had a higher educational level, and were 50-59 years of age (Table 2). The opportunistic screening rates exhibited fluctuations but without significant trends (Table 3). From 2009 to 2018, opportunistic screening rates remained relatively stable, followed by a sharp, non-significant increase in the subsequent period. The rate of opportunistic screening reached 22.7% in 2021 (Supplementary Material 1). In contrast to the organized screening rate, the opportunistic screening rate was much higher among people with higher educational levels and income across the 13 years of the survey.

### Inequality in colorectal cancer screening

**Figure 2.**
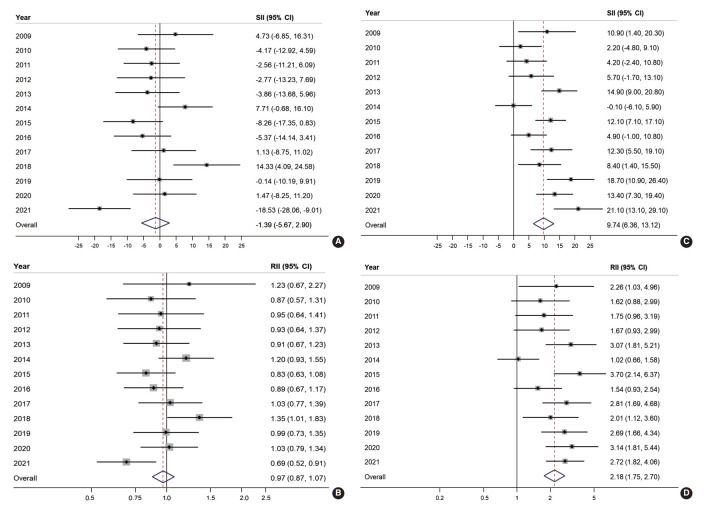
Absolute and relative educational inequalities in organized (A, B) and opportunistic (C, D) colorectal cancer screening from 2009 to 2021. SII, slope index of inequality; RII, relative index of inequality; CI, confidence interval.

Educational inequality in organized and opportunistic screening is presented in Figure 2. The SII and RII of organized screening were largely non-significant throughout the majority of the observed years. However, educational inequality was noted in opportunistic screening for approximately two-thirds of the study years. The SII of organized screening showed a negative value in 10 years out of the 13 years of the study, with an overall SII of -1.39% (95% CI, -5.67 to 2.90; Figure 2A). Similarly, the overall RII of organized screening was estimated at 0.97 (95% CI, 0.87 to 1.07; Figure 2B). In contrast, a positive SII of opportunistic screening in CRC was reported in 12 years out of 13 years of the study, with an overall estimate of 9.74% (95% CI, 6.36 to 13.12; Figure 2C). Accordingly, the overall RII of opportunistic screening in terms of education was 2.18 (95% CI, 1.75 to 2.70; Figure 2D).

**Figure 3.**
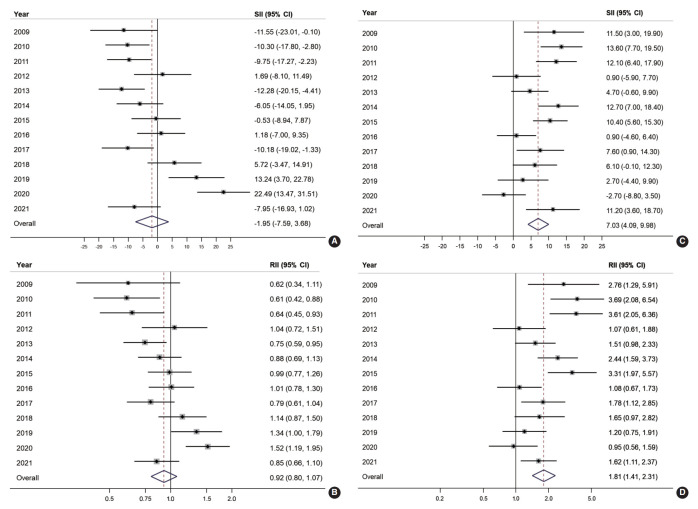
Absolute and relative income inequalities in organized (A, B) and opportunistic (C, D) colorectal cancer screening from 2009 to 2021. SII, slope index of inequality; RII, relative index of inequality; CI, confidence interval.

Figure 3 illustrates the absolute and relative income inequalities in CRC screening. The SII of organized CRC screening was mostly negative before 2015, but the pattern changed to positive starting in 2018 and then increased to 22.5% in 2020 (Figure 3A). Over the years, the overall SII in organized CRC screening was -1.95% (95% CI, -7.59 to 3.68). The RII of organized screening showed an increasing trend. It was below 0.7 during 2009-2011, then gradually rose toward 1 between 2012 and 2017, and then exceeded 1 in the following three years (2018-2020; Figure 3B). The overall RII for income inequality in organized CRC screening was 0.92 (95% CI, 0.80 to 1.07; Figure 3B). On the other hand, a positive SII was frequently observed in the opportunistic screening, with an overall SII of 7.03% (95% CI, 4.09 to 9.98; Figure 3C). Regarding income inequality, people in the highest income group were 1.81 times more likely to undergo opportunistic screening (RII, 1.81; 95% CI, 1.41 to 2.31; Figure 3D).

## DISCUSSION

The current study reported a significantly increasing trend in the overall CRC screening rate, primarily attributed to the rise in the rate of organized screening. An increasing trend in organized screening was evident across all subgroups. However, there was no notable increase in opportunistic screening over time. While there have been instances of educational and income inequality in organized screening in certain years of our study, no significant inequality was detected in the overall estimate, in either absolute or relative terms. In contrast, SES inequality was frequently noted in opportunistic screening, where individuals with higher income or education levels were more likely to participate in this type of screening.

A recent study on inequality in CRC screening, where organized screening is widely available, found that most Europeans did not exhibit significant inequality in up-to-date CRC screening [24]. The same study also indicated that Southern Europe has a high availability of opportunistic screening, which leads to SES inequality in CRC screening [24]. Suh et al. [14] reported income inequality in Korea, where individuals in the lower-income group were less likely to undergo colonoscopy screening, which is primarily opportunistic. In addition, Kim & Hwang [25] used data from the Korean National Health and Nutrition Examination Survey to report a significant reduction in inequality in CRC screening in Korea following the introduction of the NCSP. However, they found that the inequality in terms of both education and income could not be completely eliminated. Mai et al. [11] reported significant educational inequality in Korea between 2005 and 2015. These studies highlight the contribution of the NCSP in reducing inequality [25], which aligns with our study results. While there was no clear trend of educational inequality in organized screening, these indices tended to increase over the years of the study for opportunistic screening. An explanation for the educational inequality in opportunistic screening could be that those with higher educational levels had better awareness of the availability and quality of screening tests for CRC [26]. Therefore, these individuals are more likely to take advantage of screening opportunities, primarily through colonoscopy, which offers better quality and longer interval times. However, income inequality was evident in the early period of our study, suggesting that higher-income groups are more likely to opt for opportunistic screening over organized screening (Figure 3). However, in the later period, income inequality seemed to shift, with inequality in organized screening increasing. This trend is also reflected in the higher AAPC in the high-income and higher educational level groups (Table 2). Therefore, policymakers should monitor the screening rate and inequality issue more closely, particularly by income group, to ensure appropriate intervention in reducing SES inequality. Screening participation is influenced not only by screening-related policies but also by other factors. In this study, the opportunistic screening rate significantly increased to 22.7% in 2021, more than 1.5 times higher than in previous years. This sharp increase can be attributed to the coronavirus disease 2019 pandemic, which led to numerous incidents and stringent social distancing measures during the same period [27]. Due to concerns about crowded places, individuals may have preferred private screening services. Consequently, the overall inequality could have been affected by the increase in opportunistic screening rates.

To reduce overall inequality, the NCSP should contemplate suitable interventions that not only enhance the quality of organized screening participants, but also reduce the prevalence of opportunistic screening. Individuals with a higher SES or elevated risk tend to favor colonoscopy due to its superior ability to detect both cancerous and precancerous lesions, which is predominantly an opportunistic screening method. Incorporating colonoscopy as the primary screening test or as an optimal risk-based approach may be an appropriate solution, as indicated by prior studies [15,26,28-30].

The current study has some limitations. First, the use of self-reported information from the survey may have introduced a recall bias. However, given that the information pertains more to general cancer screening history rather than specific clinical details, the self-reported CRC screening data should align with medical records [31]. Second, the measurement error of income data obtained through the survey could undermine the validity of our findings regarding income inequality, primarily due to potential cognitive challenges related to income reporting, individual biases, and the presence of random errors [32]. Lastly, the use of the SII and RII to measure inequality necessitates the use of hierarchically ordered variables. As a result, we only analyzed income and education. Despite these limitations, our study offers an up-to-date and comprehensive perspective on CRC screening and associated inequalities, using high-quality data sources specifically designated for cancer screening. Therefore, the evidence provided by our study is both highly accurate and generalizable.

In conclusion, the introduction of the NCSP for CRC effectively increased the participation rate, regardless of the SES of the individuals throughout the study period. However, significant inequalities were observed in opportunistic screening related to education and income, indicating a need for improvement in overall equity in CRC screening. Future research should persistently monitor SES disparities to identify suitable interventions.

## Figures and Tables

**Figure f4-epih-45-e2023086:**
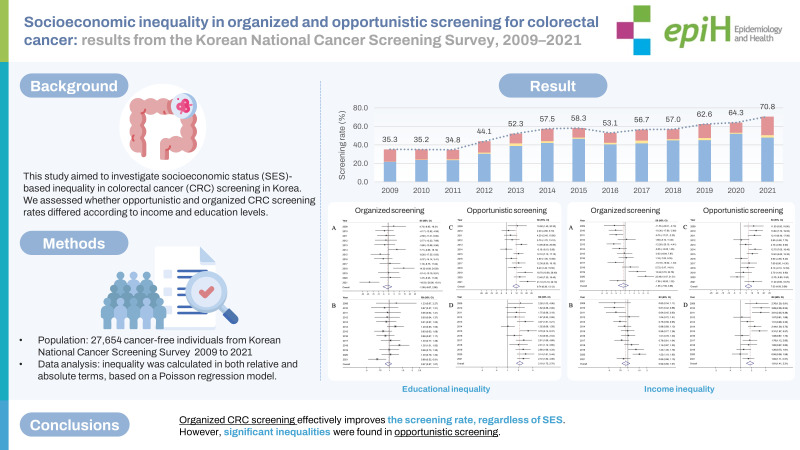


**Table 1.** Baseline characteristics of the study population in the Korean National Cancer Screening Survey (2009-2021)

Characteristics		2009	2010	2011	2012	2013	2014	2015	2016	2017	2018	2019	2020	2021
Total (n)^1^		937	1,998	2,101	2,112	2,154	2,122	2,122	2,206	2,245	2,277	2,425	2,530	2,425
Sex														
	Male	47.9	48.4	48.6	48.7	48.6	49.0	49.1	49.2	49.3	49.3	48.7	48.8	49.4
	Female	52.1	51.6	51.4	51.3	51.4	51.0	50.9	50.8	50.7	50.7	51.3	51.2	50.6
Age (yr)														
	50-59	53.5	54.6	56.1	56.2	56.7	55.9	55.0	54.4	53.7	52.9	47.3	45.8	48.5
	60-69	34.0	33.1	31.7	31.5	31.9	31.7	32.9	34.1	34.9	35.8	33.4	34.7	39.5
	≥70	12.5	12.3	12.2	12.3	11.3	12.4	12.1	11.5	11.4	11.3	19.3	19.5	12.0
Residential area														
	Metropolitan	46.1	44.0	44.9	44.3	44.4	44.9	45.6	44.1	44.9	44.2	43.8	45.3	43.1
	Non-metropolitan	53.9	56.0	55.1	55.7	55.6	55.1	54.4	55.9	55.1	55.8	56.2	54.7	56.9
Education level														
	Elementary or lower	30.1	15.8	15.2	17.9	9.8	8.2	8.2	6.8	7.6	6.3	6.5	6.7	5.4
	Middle school graduates	19.2	19.7	18.7	15.3	12.1	11.6	13.7	9.9	16.2	14.2	17.1	14.2	11.3
	High school graduates	37.9	50.6	51.5	52.6	54.9	56.7	57.1	56.4	57.6	58.9	58.6	60.4	57.2
	College/University or higher	12.8	13.9	14.6	14.2	23.3	23.5	21.0	27.0	18.6	20.6	17.7	18.6	26.1
Household income														
	Low	29.6	28.5	27.0	26.4	27.4	27.9	30.3	28.6	32.1	31.1	32.8	32.7	38.1
	Middle	36.6	36.2	37.2	41.3	32.6	37.0	36.3	41.2	40.9	34.2	32.6	32.0	27.1
	High	33.8	35.4	35.8	32.2	40.0	35.1	33.3	30.3	27.0	34.7	34.6	35.3	34.8

Values are presented as %.

1 Number is presented as weighted frequency.

**Table 2.** Organized screening rates (%) for colorectal cancer according to socioeconomic status in the Korean National Cancer Screening Survey (2009-2021)^1^

Variables		2009	2010	2011	2012	2013	2014	2015	2016	2017	2018	2019	2020	2021	AAPC^2^
Total		22.1	24.3	24.3	31.7	41.8	44.3	48.0	42.5	45.4	46.2	45.2	53.1	50.6	8.6
Sex															
	Male	20.9	24.7	23.7	31.7	40.7	47.4	50.2	43.5	46.1	47.1	45.0	54.7	50.0	8.3
	Female	23.2	23.9	24.9	31.6	42.9	41.2	45.9	41.4	44.7	45.3	45.4	51.6	51.2	8.3
Age (yr)															
	50-59	20.6	20.9	20.5	29.1	41.3	42.9	47.0	40.9	43.6	45.8	43.4	51.8	46.2	9.8
	60-69	24.1	28.7	28.5	34.9	42.3	47.1	52.1	43.9	48.1	48.0	49.1	56.2	55.3	7.1
	≥70	23.3	27.5	31.3	35.1	42.7	43.0	41.2	45.7	45.7	42.6	43.0	50.9	52.8	7.4
Residential area															
	Metropolitan	22.7	25.5	25.3	28.2	42.0	45.3	51.2	39.7	39.3	48.1	49.5	51.6	48.6	7.7
	Non-metropolitan	21.6	23.4	23.5	34.4	41.6	43.4	45.3	44.7	50.4	44.7	41.9	54.4	52.1	8.9
Education level															
	Elementary or lower	22.4	27.2	27.7	36.8	42.2	43.9	49.9	44.5	52.1	32.6	42.1	44.6	58.8	7.2
	Middle school graduates	25.0	27.6	27.5	28.5	47.9	38.2	49.4	47.0	43.1	42.8	43.4	49.8	54.6	5.0
	High school graduates	20.3	23.5	24.1	32.2	40.9	44.9	48.5	41.7	46.7	47.5	46.3	55.0	53.9	9.6
	College or higher	22.5	19.0	17.5	26.6	40.5	45.9	45.1	41.9	40.6	49.2	44.6	52.7	39.9	9.1
Household income															
	Low	24.5	30.8	31.1	34.8	44.9	46.5	48.9	44.5	50.9	41.6	42.3	48.5	55.3	6.2
	Middle	23.9	22.3	22.8	31.2	44.9	44.1	47.8	40.4	43.1	49.1	45.1	52.1	52.1	9.0
	High	18.0	21.1	20.8	29.7	37.2	42.6	47.4	43.4	42.3	47.5	48.1	58.3	44.3	10.0

AAPC, average annual percent change.

1 Screening with recommendation was defined as fecal immunochemical test within past year or colonoscopy within past 10 years.

2 AAPC is significantly different from zero at an alpha level=0.05.

**Table 3.** Opportunistic screening rates (%) for colorectal cancer according to socioeconomic status in the Korean National Cancer Screening Survey (2009-2021)^1^

Variables		2009	2010	2011	2012	2013	2014	2015	2016	2017	2018	2019	2020	2021	AAPC
Total		13.5	11.5	11.2	13.7	13.4	15.4	11.6	12.5	15.0	12.0	17.4	12.2	22.7	4.2
Sex															
	Male	15.5	14.4	13.7	15.6	14.6	16.4	14.1	12.6	18.9	13.9	18.8	13.6	27.0	4.6
	Female	11.7	8.7	8.9	11.8	12.4	14.4	9.2	12.4	11.2	10.2	16.1	10.9	18.5	4.1
Age (yr)															
	50-59	13.6	12.7	12.4	14.2	12.3	15.0	12.4	12.8	16.7	12.9	17.8	12.5	26.2	5.6^2^
	60-69	13.7	9.7	10.3	14.5	15.2	15.5	10.4	12.7	12.7	11.1	17.1	12.8	20.6	3.6
	≥70	12.9	10.6	8.2	9.1	14.3	16.7	11.5	10.5	14.3	10.9	16.9	10.3	15.5	2.6
Residential area															
	Metropolitan	12.7	12.4	12.4	15.2	14.7	16.5	11.2	13.9	13.4	12.0	16.6	13.4	25.0	4.6
	Non-metropolitan	14.2	10.7	10.3	12.5	12.4	14.5	12.0	11.5	16.3	12.1	18.0	11.3	21.0	4.3
Education level															
	Elementary or lower	9.6	6.5	5.9	11.4	12.7	14.5	4.5	8.5	8.3	5.6	6.9	7.4	14.5	2.2
	Middle school graduates	12.8	11.3	10.1	11.5	7.7	15.9	7.6	13.7	9.6	9.2	14.0	10.0	13.9	1.2
	High school graduates	12.6	10.5	10.6	13.0	13.1	14.8	10.1	11.7	14.5	12.0	18.0	11.0	19.0	3.6
	College or higher	26.6	21.0	20.5	21.2	17.6	16.9	21.0	14.7	23.9	16.2	22.4	19.6	36.3	4.3
Household income															
	Low	8.6	7.1	6.6	12.6	11.2	11.6	7.1	11.6	11.1	9.0	16.0	11.0	15.6	4.6
	Middle	12.5	8.9	8.8	11.9	12.9	14.1	10.1	12.3	14.8	12.3	16.2	11.8	24.3	6.5
	High	18.9	17.6	17.3	16.8	15.4	19.7	17.4	13.6	19.9	14.4	19.8	13.7	29.1	3.5

AAPC, average annual percent change.

1 Screening with recommendation was defined as fecal immunochemical test within past year or colonoscopy within past 10 years.

2 AAPC is significantly different from zero at an alpha level=0.05.

## SUPPLEMENTARY MATERIALS

Supplement Material 1. Trends in organized (A) and opportunistic (B) colorectal cancer screening from 2009 to 2021.
