# Supplementary material for: Socioeconomic inequality in organized and opportunistic screening for colorectal cancer: results from the Korean National Cancer Screening Survey, 2009-2021
Source: Epidemiol Health. 2023 Sep 17;45:e2023086. doi: 10.4178/epih.e2023086 (PMC10728610; doi:10.4178/epih.e2023086)
Supplement: Supplement Material 1. — Trends in organized (A) and opportunistic (B) colorectal cancer screening from 2009 to 2021. [file epih-45-e2023086-Supplementary-1.pptx]

## Slide 1
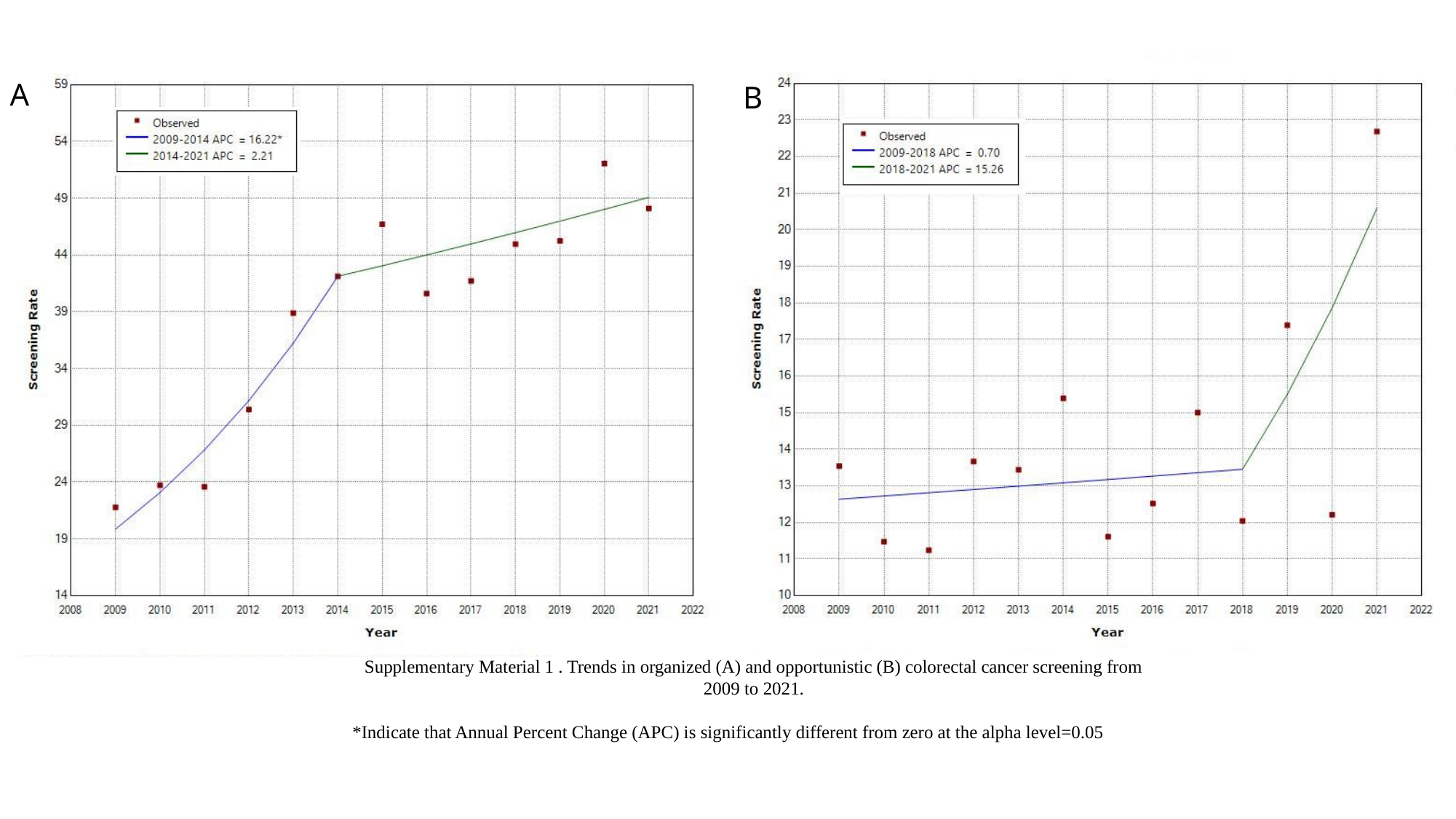

A
B
Supplementary Material 1 . Trends in organized (A) and opportunistic (B) colorectal cancer screening from 2009 to 2021.
*Indicate that Annual Percent Change (APC) is significantly different from zero at the alpha level=0.05
